# A Systematic Review of Interventions to Promoting Psychosocial Well-Being and Empowerment of Women victim of gender violence

**DOI:** 10.1192/j.eurpsy.2025.2400

**Published:** 2025-08-26

**Authors:** M. Cruz, H. Pereira

**Affiliations:** 1 Psychology, UBI, Covilhã, Portugal

## Abstract

**Introduction:**

Gender-based violence (GBV), defined as violence directed at an individual based on their gender, encompasses a range of harmful behaviors, including physical, psychological, and sexual abuse, often perpetrated by intimate partners. In this context psychosocial well-being is often severely compromised (Choden et al., 2022), and is essential interventions that can help women rebuild their sense of self, foster resilience, and improve overall mental health. Empowerment, in the context of GBV, involves not only psychological, emotional and cognitive processes in which the person is able to think positively about themselves and their abilities and master individual and social aspects of their life, but also social and relational processes, since violence occurs in relationships with others (Amirroud et al., 2022), and therefore, empowerment allows women to assert their rights, build confidence, and engage actively in social and economic activities (Malhotra & Schuler, 2005).

**Objectives:**

This systematic literature review (SLR), based on the PRISMA 2020 guidelines, aims to provide an updated overview of interventions designed to promote psychosocial well-being and/or empowerment (PWE) among women who are victims of gender-based violence, with the goal of informing future intervention projects.

**Methods:**

Data collection was conducted through the SCOPUS and Web of Science databases, focusing on studies published between 2018 and January 15, 2024, in English. The inclusion and exclusion criteria were based on the PICO framework: (P) women affected by gender-based violence, (I) interventions to enhance PWE, (C) comparison between initial and final intervention phases, and (O) assessed outcomes related to PWE. thirteen studies were included.

**Results:**

The primary intervention components were counseling, health education, mindfulness and relaxation practices and expressive therapies. Experimental studies reported significant improvements in mood, reductions in depressive symptoms, anxiety, and stress levels. Empowerment components were less frequently addressed, they also showed positive outcomes, including increases in self-esteem and self-efficacy. Overall, EG demonstrated superior results across nearly all assessed variables compared to CG. The most effective interventions were those incorporating mindfulness and relaxation, counseling techniques, and expressive strategies. Key limitations of the included studies involved methodological quality, sample size, and representativeness, as well as the potential for response bias.

**Conclusions:**

This SLR offers an original contribution by advocating for evidence-based practice and providing valuable insights for healthcare professionals, policymakers, and researchers engaged in the integrative health care of women impacted by gender-based violence.

**Disclosure of Interest:**

None Declared

